# Factors Affecting the Regeneration, via Organogenesis, and the Selection of Transgenic Calli in the Peach Rootstock Hansen 536 (*Prunus persica* × *Prunus amygdalus*) to Express an RNAi Construct against PPV Virus

**DOI:** 10.3390/plants8060178

**Published:** 2019-06-17

**Authors:** Silvia Sabbadini, Angela Ricci, Cecilia Limera, Dania Baldoni, Luca Capriotti, Bruno Mezzetti

**Affiliations:** Department of Agricultural, Food and Environmental Sciences, Marche Polytechnic University, 60131 Ancona, Italy; s.sabbadini@staff.univpm.it (S.S.); angela.ricci@pm.univpm.it (A.R.); c.o.limera@pm.univpm.it (C.L.); dania.baldoni@gmail.com (D.B.); l.capriotti@pm.univpm (L.C.)

**Keywords:** *Prunus* spp., organogenesis, genetic transformation, GFP, RNAi, PPV

## Abstract

*Prunus* spp. is one of the most recalcitrant fruit tree species in terms of in vitro regeneration and transformation, mostly when mature tissues are used as explants. The present study describes the in vitro regeneration via indirect organogenesis, and *Agrobacterium tumefaciens*-mediated transformation of the peach rootstock Hansen 536 (*Prunus persica* × *Prunus amygdalus*) through the use of meristematic bulks (MBs) as starting explants. Efficient adventitious shoot regeneration was obtained when Hansen 536 MBs were cultured on an optimized medium consisting of modified McCown Woody Plant medium (WPM) enriched with 4.4 μM 6-Benzyladenine (BA), 0.1 μM 1-Naphthaleneacetic acid (NAA) and 6.0 g L^−1^ plant agar S1000 (B&V). MB slices were used later as starting explants for *Agrobacterium*-mediated transformation to introduce an RNAi construct “ihp35S-PPV194” against PPV virus. Transgenic events were identified by both green fluorescent protein (GFP) screening and kanamycin selection at different concentrations (0, 17 or 42 μM). GFP-fluorescent proliferating callus lines were selected and confirmed to stably express the *ihp35S-PPV194::eGFP* gene construct by molecular analysis. Although shoot regeneration from these transgenic calli has not been obtained yet, this represents one of the few examples of successful attempts in peach genetic transformation from somatic tissues, and also serves as a useful in vitro system for future gene functional analysis in peach.

## 1. Introduction

Traditional breeding techniques applied in the genetic improvement of *Prunus* species, especially peach and nectarines, are characterized by a significant level of heterozygosity. Additionally, laborious and time-consuming backcrossing cycles are a challenge, especially since agronomical traits are often recessive and/or controlled by several different genes [1,2]. Virus resistance induction is among the primary goals in plant genetic improvement, and new breeding techniques (NBTs) represent a promising integrative tool to traditional methods, especially when applied on woody fruit species such as *Prunus* spp. [3,4,5,6,7,8,9,10]. Since its first discovery, RNA Interference (RNAi) has become one of the most promising new strategies applied to induce resistance in plants against different kinds of pathogens, including viruses, fungi, and insects [11,12,13,14]. This mechanism is known to regulate endogenous gene expression and has been discovered as a natural conserved defense strategy used by plants against virus infections [15,16,17,18]. It relies upon the production of double-strand RNAs precursors, which targets complementary transcripts in a sequence-specific manner, leading to the mRNA degradation or translation inhibition. The silencing molecules at the basis of RNAi mechanism has demonstrated to be capable of moving through the plant cell plasmodesmata or plant vascular system, for both short and long distances, respectively [19,20,21]. In the case of woody plants species, this systemic transfer of silencing signal represents an advantage also when considered from a biosafety perspective, because the introduction of RNAi-based gene constructs directly in a rootstock, is expected to result in the transport of the silencing molecules to the non-modified scion (trans-grafting) [5,6,10,22,23]. However, the application of these techniques still often relies on the stable introduction of the gene of interest into the plant genome, which presents a challenge for woody plant species as they are characterized by a high level of recalcitrance to transformation. Indeed, for an efficient gene introgression into the host genome, different parameters need to be considered and optimized, such as: (i) choice of starting plant material; (ii) in vitro regeneration medium composition (i.e., salt composition of basal medium, plant growth regulators combination, the gelling agent and the optional addition of antioxidant compounds, ethylene inhibitors, etc.); (iii) the bacterial strain chosen as vector, and the antibiotics used to contain bacterial persistence in the medium post transformation (when *Agrobacterium*-mediated transformation is exploited), and (iv) selection method applied to isolate putatively transformed lines and curbing the regeneration of escapes (non-transgenic shoots) [3,24,25,26]. Different successful in vitro regeneration protocols have been developed for *Prunus* species, particularly in peach, using different starting tissues; among these reports, the majority used immature seeds and cotyledons as starting explants [27,28,29,30,31]. However, regeneration from adult tissues is more recommended for clonally propagated crops to retain their desirable characteristics, especially for those with high heterozygosity as peach × almond hybrid, usually commercially used for peach plant propagation. In vitro shoot regeneration and/or callus induction from somatic tissues has been obtained in different peach genotypes starting from in vitro leaves [32,33,34,35], stems, petioles and flower calyx [36]. Furthermore, a protocol developed by us on table grape [37] has been effectively utilized also for the in vitro regeneration via organogenesis, and genetic engineering of different peach varieties and rootstocks [38,39]. It is characterized by the induction of MB formation starting from apices of in vitro cultured shoots; the MBs obtained are characterized by high competence for regenerating new shoots, providing the basis for a useful tool to genetically transform peach starting from adult tissues. A previous study carried out by us on the genetic transformation of the peach rootstock GF677 (*Prunus persica* × *Prunus amygdalus*) demonstrated the difficulty of transforming MBs belonging to this hybrid, indeed a transformation efficiency of 0.3% was achieved, which led to the obtainment of two independent transgenic lines expressing an empty vector, named as hp-pBin19 [40].

In the present study, we aimed to optimize the regeneration protocol, via organogenesis, and *Agrobacterium*-mediated transformation of another commercial peach rootstock, Hansen 536 (*Prunus persica* × *Prunus amygdalus*) to introduce a hairpin gene construct against Plum pox virus (PPV), which causes Sharka disease in several *Prunus* species [8].

## 2. Results

### 2.1. Influence of Basal Media, Growth Regulators and Gelling Agents on Regeneration Efficiency of MBs

Eighteen combinations consisting of different basal media and plant growth regulators (PGRs) were applied to increase regeneration efficiency of Hansen 536 MBs. Generally, the results obtained showed higher regeneration efficiency, when Quoirin & Lepoivre medium (QL) and modified WPM (WPMm) were used as basal media (Table 1). Results also presented some optimal combinations, particularly media QL2, QL6 and WPMm5 showed higher values in terms of regeneration frequency, i.e., 83.3%, 83.3% and 77.8%, respectively. These combinations contained either QL or WPMm as basal media, enriched with BA combined with either NAA or Indole-3-butyric acid (IBA). On the contrary, except for medium DKW/Juglans six (DKW6), all PGRs combinations used with DKW as basal medium reported lower efficiency. A widespread problem of hyperhydricity was observed in the culture media tested, which were all supplemented with plant agar (PA) as gelling agent. Thus, a second regeneration trial was carried out with the main aim of reducing vitrified shoots, by adding to media QL2, QL6 and WPMm5, 6 g L^−1^ B&V in comparison to the addition of 7 g L^−1^ PA. Results showed a significant increase in regeneration efficiency (mean number of regenerating shoots per explant) only in medium WPMm5 when MB slices were cultured on media with B&V instead of PA (Table 2). While, the frequency of vitrified shoots decreased significantly for all the three media combinations when explants were placed on media containing 6 g L^−1^ B&V; medium WPMm5 showed the best results. For this reason and considering the high regeneration efficiency obtained from the two trials, medium WPMm5 (modified WPM, 4.4 μM BA, 0.1 μM NAA, 6.0 g L^−1^ B&V agar) was chosen as regeneration medium for the transformation experiment in this study.

### 2.2. Hansen 536 MBs Transformation Efficiency and the Role of GFP and Kanamycin as Selectable Markers

**Figure 1:**
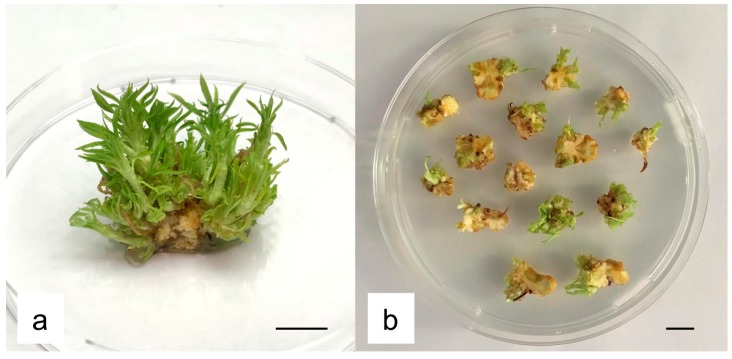
Hansen 536 regeneration via organogenesis: (**a**) adventitious shoot regeneration after 30 days of culture on WPMm5 medium. (**b**) MB slices (1 cm^2^, 2 mm thick) used as starting explant for the transformation experiment (bar = 1 cm).

**Figure 2:**
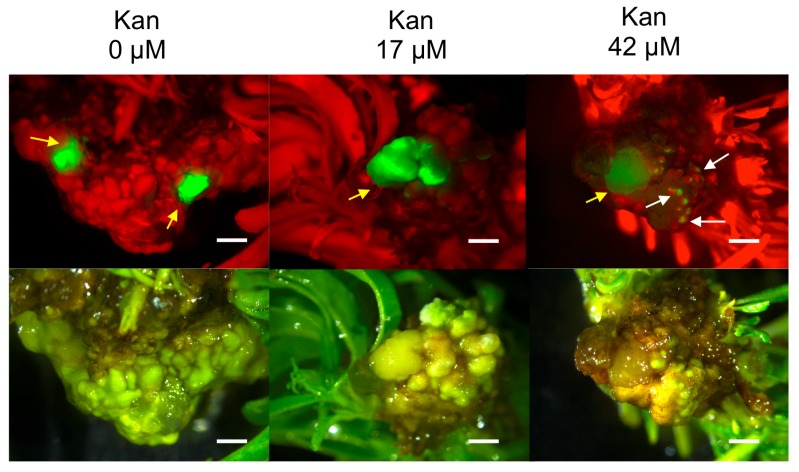
Hansen 536 GFP fluorescing spots and/or Lzs detected on MB slices at three weeks after co-culture on medium with 0, 17 or 42 μM Kan. White and yellow arrows indicate examples of GFP spots and large zones of fluorescent actively growing calli, respectively. Uniform bright green fluorescence was observed under UV light. Upper and lower panels show images taken under UV and white light, respectively (bar = 2 mm).

**Figure 3:**
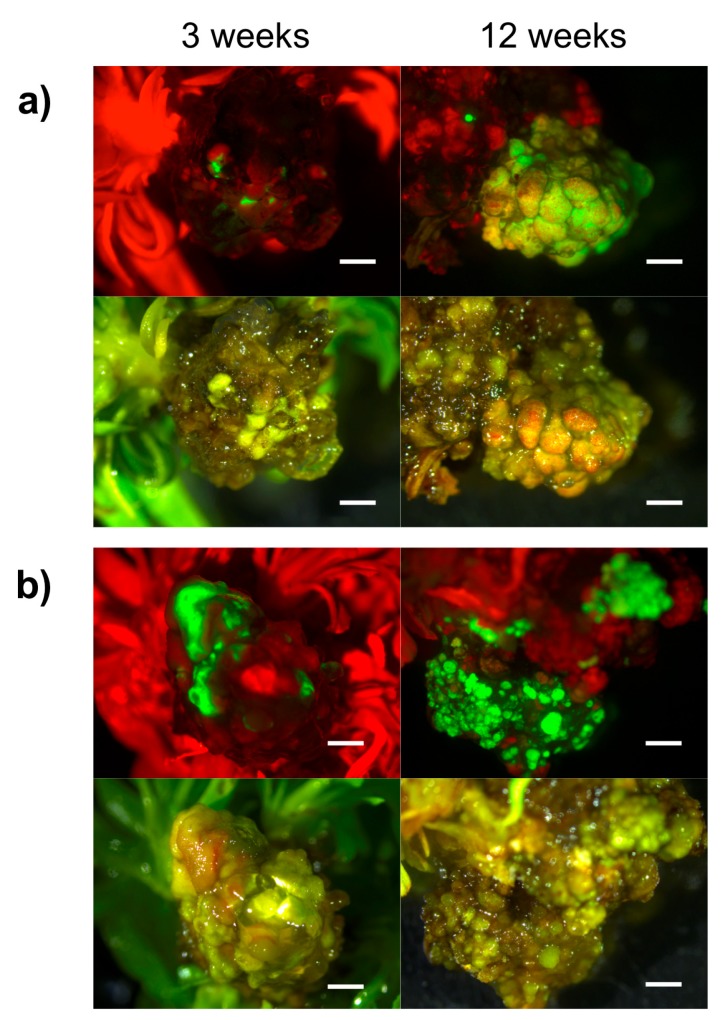
Hansen 536 GFP fluorescing spots and/or Lzs detected on MB slices at three weeks and 12 weeks after co-culture on medium with 17 μM Kan: (**a**,**b**) MB slices obtained from two independent MBs presenting Lzs of GFP expression actively growing within 12 weeks of culture. Uniform bright green fluorescence was observed under UV light. Upper and lower panels show images taken under UV and white light, respectively (bar = 2 mm).

MB slices obtained from 30-day-old MBs cultured on medium WPMm5 were used as starting explants for *Agrobacterium*-mediated transformation trial (Figure 1). In a previous study carried out by us, we investigated the sensitivity of untransformed Hansen 536 MBs to kanamycin by placing the explants on WPMm5 medium with different kanamycin (Kan) concentrations (from 0 up to 117 μM); the results obtained showed that Kan concentrations higher than 50 μM extremely inhibited the in vitro regeneration of Hansen 536 untransformed MBs, hence leading to the complete necrosis of the explants after six weeks of culture (data not shown). Thus, lower concentrations of Kan (0, 17 or 42 μM) were tested in this study to allow the survival of putatively transformed cells and the eventual possibility of regenerating transgenic shoots. The influence of Kan on the frequency of GFP fluorescing explants was investigated. The number of Hansen 536 MB slices with at least one fluorescing spot and the presence of actively growing calli showing GFP were annotated at three and 12 weeks after co-culture on medium WPMm5 enriched with 0, 17 or 42 μM Kan (Table 3). The results obtained showed significant differences, related to the culture conditions applied (i.e., 0, 17 or 42 μM Kan), in terms of both percentage of MB slices expressing GFP and mean number of GFP spots per MB slice at three weeks after co-culture. The higher value (3.17) with regard to the mean number of GFP spot/explant was recorded for the MB slices cultured on 42 μM Kan, while the highest value (about 63%) in terms of percentage of GFP fluorescing MB slices was obtained on 17 μM Kan (Table 3); in contrast, no statistical differences were observed at 12 weeks after co-culture. Results related to the percentage of MB slices presenting Large coalesced zones (Lzs) expressing GFP at each culture condition presented better results (about 37 %) when explants were placed on media with 42 μM Kan at three weeks after co-culture. In particular, a total number of 12, 15 and 22 MB slices (calli derived from different starting MBs) presenting Lzs of GFP expression were selected at 0, 17 and 42 μM Kan, respectively (Table 3 and Figure 2). While, at 12 weeks after co-culture, explants placed on 17 μM Kan showed higher values compared to the other two Kan concentrations. Indeed, about 6% of MB slices presented Lzs of GFP expression actively growing on 17 μM Kan (Table 3 and Figure 3a,b), while 3% and 1% of MB slices with Lzs expressing GFP were obtained on 0 and 42 μM Kan, respectively (Table 3). In particular, the absence of kanamycin selection in the regeneration medium led to a major production of chimeric calli, in which the containment of non-transformed calli sections was challenging, and often the latter prevailed over the transformed ones over time; on the contrary, explants cultured in the presence of 42 μM Kan tended to necrotize during the 12 weeks after co-culture.

### 2.3. Molecular Analysis of Putatively Transgenic Callus Lines

**Figure 4:**
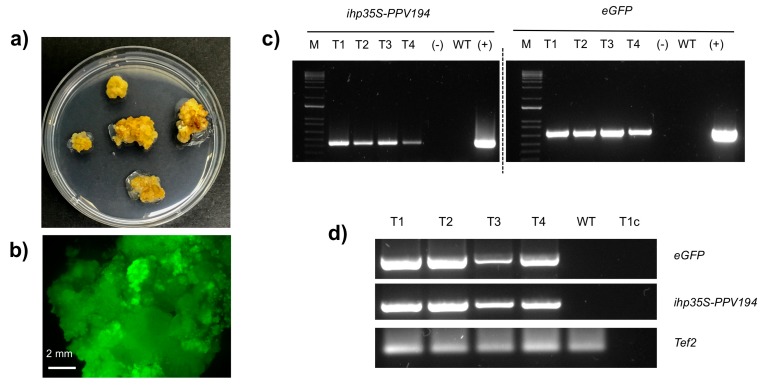
Hansen 536 transgenic callus lines and molecular analysis of transformants: (**a**) Callus line proliferated for five months after *Agrobacterium* infection. (**b**) Transgenic callus expressing uniform GFP fluorescence. (**c**) Amplification of *ihp35S-PPV194* (456 bp) and *eGFP* (701 bp) genes fragments from the genomic DNAs of four transgenic (T1-T4) and one wild-type (WT) callus lines obtained from different MBs, and *pK7WG2-ihp35S-PPV194::eGFP* “(+)”. The lane labelled “(−)” shows the PCR result using water as negative control. M, DNA marker (1Kb Plus DNA Ladder, Invitrogen, Carlsbad, CA, USA). (**d**) RT-PCR analysis of *eGFP* (701 bp), *ihp35S-PPV194* (456 bp) sequences of four transgenic (T1-T4) and one wild-type (WT) callus lines obtained from different MBs. The 129 bp fragment of cDNA from housekeeping gene *Tef2* was amplified as control to validate RT-PCR results. The lane labelled “T1c” shows the PCR result using total RNA of the line T1 as template control (RNA sample treated with DNase but without reverse transcription was used as PCR template to evaluate DNA contamination).

Twelve weeks after co-culture, there were no transgenic shoots regenerated from Lzs detected, but stable GFP expressing callus lines (calli derived from different starting MBs) (Table 3) were isolated, divided into small pieces and placed on fresh WPMm5 medium for proliferation with the corresponding Kan concentrations (i.e., 0, 17 or 42 μM Kan). One to three grams of calli were generated after two months of culture (with monthly sub-culture) from each GFP fluorescing callus line (Figure 4a,b), which provided sufficient material for molecular analyses. Total genomic DNA and RNA were extracted from one wild-type callus line and four GFP expressing callus lines, two from those proliferating on 17 μM Kan, one proliferating on 42 μM Kan and one from those proliferating in 0 μM of Kan in the medium, respectively. The insertion and the transcription of *eGFP* gene and ihp35S-PPV194 sequence into the callus lines genomes were checked by PCR and RT-PCR analysis. 701 bp of the *eGFP* coding sequence and 456 bp of the ihp35S-PPV194 sequence were successfully amplified from the four GFP fluorescing callus lines analysed, confirming their transgenic state and the stable expression of the *ihp35S-PPV194::eGFP* gene construct at five months after *Agrobacterium* infection (Figure 4c,d).

## 3. Discussion

There are few reports describing successful methods for the regeneration of adventitious shoots in peach when using adult tissues as starting plant material, and a few of them are focused on the use of MBs as efficient starting explant for shoot regeneration from both peach rootstocks (Garnem and GF677) and scions (UFO-3, Maruja, Flariba and Alice Bigi) [38,39,40]. The MB has been described as a cluster of cells able to continuously regenerate new adventitious buds distinguishable at the surface of the callus [37]; histological studies showed that, internally, MB is composed of hypertrophied parenchymatic cells and initiation nodules from which the adventitious shoots originate [37]. Several studies confirmed the versatility of this regeneration protocol, also for the in vitro organogenesis of plant species other than peach [41,42,43]; however, its optimization is required for the individual genotypes under study, that often showed different levels of reaction to external induction treatments in regards to in vitro morphogenesis [39,43]. It has been reported that several of the differences observed in the organogenetic response, even within genotypes of the same species, greatly depend on the level of reaction to PGRs signals, which can vary also on the basis of the starting explant used [32,44,45]. The use of different basal salts tested in our study, proved that QL and WPMm were more appropriate combinations of micro and macro salts for regeneration of Hansen 536 MB slices. In particular, our investigation showed a positive effect on shoots regeneration of Hansen 536 when QL or WPMm were supplemented with the cytokinin BA combined either with NAA or IBA; similar results were reported by other authors working on organogenesis of *Prunus* spp. and also of other fruit species [33,36,39,46,47,48,49,50]. The effect of gelling agents on Hansen 536 MB slices was also investigated. The strong influence that composition and concentration of gelling agents have in regard to shoot hyperhydricity and plant regeneration efficiency in woody fruit tree species has been well documented [48,51,52,53]. Our results indicated that, the use of B&V as the gelling agent had a positive effect on Hansen 536 regeneration, as well as reduction of vitrified shoot percentage, compared to PA agar. Even though, the composition and gel strength of the two agars used are quite similar, they led to significantly different results, especially concerning hyperhydricity effect on Hansen 536 shoots. Furthermore, the combination of B&V agar with medium WPMm5 induced a significant decrease in the percentage of vitrified shoots compared to the other basal media and PGRs combinations tested (media QL2 and QL6) (Table 2). This revealed that, several factors other than gelling agents have a role on hyperhydricity and shoot regeneration. Several studies have reported on the role of single ions present in culture media, such as NH_4_, NO_3_^−^ and Ca^2+^, on in vitro plant culture in relation with hyperhydricity [54,55,56]. In particular, Alanagh et al (2014) [54], found that a low concentration of Ca^2+^ (lower than 3 mM) combined with a medium concentration of NO_3_^-^ (between 7–21 mM) is recommended for the proliferation of healthy shoots of peach rootstock GF677. Medium WPMm5 used in our investigation contained 16.86 mM of NO_3_^−^ combined to 6.6 mM of Ca^2+^, in comparison with medium QL2 and QL6, which are characterized by a lower Ca^2+^ concentration (3.53 mM) with almost double content of total NO_3_^−^ (29.88 mM). This higher content of NO_3_^−^ could explain the higher number of vitrified shoots obtained in our study when MB slices were cultured on media QL2 and QL6.

The optimized regeneration medium was utilized for subsequent genetic transformation trial with the hairpin gene construct ihp35S-PPV194, which also achieved selection of putative transgenic lines through the two marker genes, *eGFP* and *nptII*. A similar hairpin sequence was already validated in a previous study carried out in *N.benthamiana* plants, demonstrating its ability in conferring systemic resistance against PPV virus [57]. With the aim to achieve stable transformation with this construct, GFP expression on proliferating cells after *Agrobacterium* infection of Hansen 536 MB slices was exploited to isolate and proliferate stable transgenic callus lines. In this regard, expression of the vital reporter gene *eGFP* was crucial to assess the progress of GFP fluorescing calli formation in the first 12 weeks after co-culture. Furthermore, it helped in evaluating the possible role of kanamycin in stabilizing transient expression in cells at three weeks after co-culture; most transgenic events are considered stable after this period [7,58]. The results obtained showed how the transformation protocol used, previously applied on GF677 MB slices [40], was also effective for the obtainment of Hansen 536 GFP fluorescing actively growing calli expressing the *ihp35S-PPV194::eGFP* construct in all the three culture conditions applied (0, 17, 42 μM Kan), with best results obtained when 17 μM Kan was supplemented in the culture medium (Figure 3 and Table 3). In addition, the combined use of GFP and kanamycin selection facilitated the early elimination of regenerating escapes (both shoots and calli sectors), which compete against transformed cells. Similar results have been reported in *Prunus* spp., including peach, and other woody plant species genetic transformation, confirming the usefulness of reporter genes and antibiotic selection in increasing the frequency of transgenic events and avoiding regeneration of escapes [7,43,59,60,61]. In this study, the selection of transgenic events, even in absence of kanamycin in the medium, was investigated; in this culture condition about 20% (at three weeks) and 3% (at 12 weeks) of MB slices with large coalesced zones of GFP expression were selected using GFP screening alone (Figure 2 and Table 3). We recently demonstrated in a similar work performed on grapevine MBs, the possibility of isolating transgenic shoots/calli lines with only the use of non-destructive marker genes, like *gfp* [42]. However, similarly to the results obtained in the present study, we observed a lower efficiency, both in terms of frequency of transgenic plants/calli lines obtained and proliferation ability of GFP expressing cells, when MB slices were cultured on non-selective conditions [42]. Indeed, in the present study the results obtained confirm the strong influence played by kanamycin in obtaining transgenic events in a more effective way, especially during the first weeks of selection (Table 3), inhibiting also the regeneration of non-transformed shoots (escapes) or the proliferation of non-transgenic calli sectors. Nevertheless, the obtainment of transgenic actively growing calli of Hansen 536 also in the absence of kanamycin in the medium at early stages of transformation, open the possibility, even though with a much lower frequency, of isolating transgenic calli exploiting only reporter genes as selective agent. This scenario would help to avoid the use of antibiotic-based selection strategies to obtain antibiotic marker-free transformed plants, reducing the risk assessment process and public concerns [62,63,64].

In conclusion, we established an efficient regeneration protocol to obtain adventitious shoots of Hansen 536 peach rootstock starting from somatic tissues; this protocol was exploited for subsequent transformation trial that led to the recovery of stable transgenic callus lines of Hansen 536 stably expressing the *ihp35S-PPV194::eGFP* gene construct. Even if the protocol needs to be optimized for inducing shoot regeneration from these transgenic callus lines, to our knowledge this represents one of the few examples, or even the first successful attempt of producing transformed peach calli, and a promising protocol to obtain regenerated shoots expressing the anti-PPV RNAi construct from peach somatic tissue. Furthermore, these in vitro proliferating transgenic calli represent a valuable tool to both explore the genetic and molecular features that hamper the regeneration of transgenic shoots and also for future functional analyses of genes and omics studies [65,66,67].

## 4. Material and Methods

### 4.1. Plant Material and Establishment of In Vitro Culture

In vitro proliferating cultures of the commercial peach rootstock Hansen 536 (*Prunus persica* × *Prunus amygdalus*) were established from shoot tips of 10-year-old Hansen 536 trees, grown in the greenhouse of Vitroplant Italia, Cesena, Italy. The sterilization protocol consisted of washing the plant materials in a solution of 1% (V/V) sodium hypochlorite for 15 min, rinsed three times with sterile water, and sub-cultured every 30 days on propagation medium. This medium is composed of MS basal salts and vitamins [68], 30 g L^−1^ sucrose, and 7 g L^−1^ plant agar (Duchefa Biochemie, Italy), with the addition of 4.4 μM BA and 0.05 μM NAA (pH 5.6–5.7).

Meristematic bulks (MBs) of Hansen 536 were induced and maintained following the protocols previously described [37,40]. Briefly, MB induction was started from shoot tips, obtained from two-year-old in vitro proliferating culture, which were topped and cultured on induction medium (IM) composed of MS microelements and vitamins, 10.40 mM KNO_3_, 5 mM NH_4_NO_3_, 1.47 mM KH_2_PO_4_, 1.62 mM MgSO_4_ ×·7H_2_O, 4.57 mM CaNO_3_, 1.67 mM NaH_2_PO_4_, 30 g L^−1^ sucrose, 7 g L^−1^ plant agar (Duchefa Biochemie, Italy), pH 5.6–5.7. The explants were sub-cultured three times on IM medium enriched with 0.05 μM NAA and increasing concentration of BA (from 4.4 μM up to 13.2 μM), by topping the apical dome at each sub-culture. MBs obtained were maintained on IM_3_ medium, which consisted of IM medium with the addition of 13.2 μM BA and 0.05 μM NAA and placed in the growth chamber at 24 ± 1 under a photoperiod of 16-h light (70 μmol/m^2^/s) provided by white fluorescent tubes.

### 4.2. Influence of Different Factors on the Regeneration Efficiency of Hansen 536 MBs

#### 4.2.1. Effect of Different Basal Media and Plant Growth Regulator Combinations

Thirty-day-old MBs of Hansen 536 cultured on IM_3_ were cut into slices (1 cm^2^, 2 mm thick) and utilized as starting explants for the regeneration experiments. MB slices were cultured in 9-cm diameter microboxes (Micropoli, IT) and used for testing 18 different media combinations, which differed in basal salts and vitamins composition: full-strength QL [69], DKW [70] and modified WPM [71]. Each of these media was supplemented with different combinations of plant growth regulators (PGRs), including BA, Thidiazuron (TDZ), NAA and Indole-3-butyric acid (IBA) (Table 4). Each culture medium also contained 7 g L^−1^ plant agar (Duchefa Biochemie, Italy), 30 g L^−1^ sucrose, and the pH was adjusted to 5.6–5.7 before autoclaving at 121 °C for 20 min. Three microboxes, containing six MB slices each, were evaluated for each medium composition. Data on the MB regeneration frequency, evaluated as [(number of MB slices regenerating at least one shoot/total MB slices treated) × 100], and on the average number of regenerating shoots per explant were acquired after 30 days.

#### 4.2.2. Effect of Different Gelling Agents on Shoot Regeneration Efficiency and Shoot Hyperhydricity 

MB slices obtained from 30-day-old MBs cultured on IM_3_ were placed on culture media containing two different commercial agar i.e., 7 g L^−1^ plant agar (Duchefa Biochemie, Italy), or 6 g L^−1^ plant agar S1000 (B&V, Italy), referred to as PA and B&V respectively in this study. These different gelling agents were combined with the components and culture condition of the media QL2, QL6 and WPMm5 described in Table 4. Data on the average number of regenerating shoots per explant and the frequency of vitrified shoots regenerating per MB slice were acquired after 30 days of culture. The two gelling agents were added at the concentration recommended by the manufacturers, and these produced culture media with similar compactness.

### 4.3. Genetic Transformation

#### 4.3.1. Gene Construct and Agrobacterium Tumefaciens Strain

In this study, a hairpin gene construct was designed to silence PPV virus, and it contains the constitutive promoter 35S (543 base-long) of CaMV (*Cauliflower Mosaic Virus*). Each arm of the hairpin structure was 194 base-long sequence, which spans from base 134 to base 327 of the PPV polyprotein (accession number X16415). The two arms were joined by 115 base-long InLAX intron of the *LAX1* gene of *Medicago truncatula*. The hairpin sequence was cloned firstly in the pbin19 binary vector [72] and secondly sub-cloned in the pK7WG2 binary vector [73], which expresses the eGFP protein, and that confers resistance to kanamycin through NosPromoter*-nptII-*NosTerminator cassette. The gene construct (*ihp35S-PPV194::eGFP*) was inserted in *A. tumefaciens* strain EHA105 by electroporation [74].

#### 4.3.2. Genetic Transformation Protocol and Selection of GFP Fluorescing Calli 

Transformation trial was carried out following the protocol previously described [40] with some modifications. Single colonies of *A. tumefaciens* strain EHA105 were inoculated in liquid YEB medium supplemented with 152 μM spectynomicin and 61 μM rifampicin to reach an OD_600_ = 1.0–1.2. The bacterial culture was pelleted and re-suspended in MS basal salt and vitamins liquid medium including 20 g L^−1^ sucrose, 100 μM acetosyringone, pH 5.2. The explants were then dipped in the infection solution for 15 min, subsequently dried on sterile filter paper, and finally placed on co-culture medium (MS basal salt and vitamins, 30 g L^−1^ sucrose, 100 μM acetosyringone and 7 g L^–1^ plant agar) for 48 h at 24 ± 1 °C in dark conditions. After co-culture, MB slices were placed on regeneration medium WPMm5 supplemented with 0, 17 or 42 μM Kan, to select putative transgenic lines and/or calli, and 473 μM carbenicillin plus 419 μM cefotaxime, used to eliminate *A.tumefaciens* contaminations.

For each of the three treatments (Kan 0, 17 or 42 μM), 12 microboxes were prepared; each containing five MB slices, for a total of 60 MB slices per treatment. Data on the mean number of GFP spot per explant and the percentage of MB slices showing GFP fluorescing calli area were acquired at three and 12 weeks after co-culture. Furthermore, large coalesced zones (Lzs) of GFP expression (actively growing calli showing GFP fluorescence), were annotated as well as the frequency of MB slices showing Lzs of GFP expression at three and 12 weeks after co-culture. GFP fluorescing calli were detected by observing the explants under the stereomicroscope Leica MZ10F (Leica Mikrosystems GmbH, Wetzlar, Germany) (λEX = 480nm and λEM = 510 nm), and by using the Leica DFC 450 C290 camera for images acquisition, which were processed through Leica Application Suite V.4.5 (Leica Microsystems GmbH, Wetxlar, Germany).

### 4.4. Molecular Analysis of GFP Fluorescing Calli

About 100 mg calli from four GFP fluorescing and one wild-type callus lines derived from different starting MBs of Hansen 536 were collected. Total genomic DNA was isolated from each callus line using the commercial kit “Nucleon Phytopure” (Amersham Bioscience) following the manufacturer instructions. PCR analyses were carried out to amplify 701 bp of the eGFP coding sequence with the following primers: eGFP-F, 5′-GTGAGCAAGGGCGAGGAG-3′ and eGFP-R, 5′-TCCATGCCGAGAGTGATCCC-3′. Furthermore, 456 bp of the *ihp35S-PPV194* gene sequence, which spans from the antisense arm of the hairpin sequence to the NOS terminator (NOS ter), were amplified with the following primers: hpPPV-F, 5′-TAGCTGTTGCACTCTCATATGTGTTT-3′ and NOSTer-R, 5′-GGAAGGGAC TGGCTGCTATTGGGCGAA-3′. Total RNA was extracted from 100 mg of the collected samples with RNeasy^®^ Plant mini Kit (Qiagen, Hilden, Germany) following the manufacturer instructions. Samples were treated with DNase to be cleaned from any genomic DNA contamination, and 1 μg of total RNA was primed with oligo(dT)_15_ and reverse transcribed using Goscript^TM^ Reverse Transcription System (Promega) following the manufacturer instructions. For the expression analysis of the *ihp35S-PPV194::eGFP* gene construct, 701 bp of the eGFP coding sequence and 456 bp of the ihp35S-PPV194 gene sequence were amplified with the same pair of primers used for PCR analysis. A fragment of 129 bp of the translation elongation factor 2 (*Tef2*) coding sequence was amplified from each sample and used as positive control [75]. The PCR conditions were as follows: 95 °C for 5 min; 40 cycles at 95 °C for 30 s, 60 °C for 30 s, and 72 °C for 1 min; and 72 °C for 5 min. The plasmid DNA of pK7WG2-ihp35S-PPV194::eGFP was used as positive control, while the DNA or cDNA from a wild-type callus line, and sample containing RNA template were used as negative controls. 10 μL of amplified fragments were loaded on agarose gel (2%, w/v) with SYBER^®^ Safe DNA Gel Stain (Invitrogen) and detected by UV after electrophoresis.

### 4.5. Statistical Analysis

The results obtained were analyzed by one-way ANOVA, and Duncan test (*p* < 0.05) was used to identify significant differences.

## Figures and Tables

**Table 1:** Regeneration efficiency obtained from Hansen 536 MB slices after four weeks of culture on different combinations of basal media and PGRs.

Regeneration Media	Frequency of Regeneration (%) ± SE *	Average Number of Regenerated Shoots/MB ± SE
QL1	61.1 ± 5.5 ^(abc)^	1.8 ± 0.5 ^(defg)^
QL2	83.3 ± 9.6 ^(a)^	6.4 ± 1.2 ^(ab)^
QL3	61.1 ± 11.1 ^(abc)^	3.8 ± 1 ^(bcde)^
QL4	50 ± 0 ^(abc)^	2.8 ± 1.9 ^(cdefg)^
QL5	66.7 ± 9.6 ^(abc)^	4.3 ± 1.2 ^(bcd)^
QL6	83.3 ± 16.7 ^(a)^	7.2 ± 1.3 ^(a)^
DKW1	38.9 ± 5.5 ^(abc)^	1.4 ± 0.6 ^(defg)^
DKW2	38.9 ± 22.2 ^(abc)^	1.3 ± 0.5 ^(defg)^
DKW3	27.8 ± 5.5 ^(bc)^	0.8 ± 0.3 ^(fg)^
DKW4	22.2 ± 11.1 ^(c)^	0.4 ± 0.2 ^(g)^
DKW5	27.8 ± 5.5 ^(bc)^	1.2 ± 0.5 ^(efg)^
DKW6	72.2 ± 14.7 ^(ab)^	3.5 ± 0.8 ^(bcdef)^
WPMm1	61.1 ± 22.2 ^(abc)^	2.6 ± 0.7 ^(cdefg)^
WPMm2	72.2 ± 11.1 ^(ab)^	5.5 ± 1.4 ^(abc)^
WPMm3	38.9 ± 5.5 ^(abc)^	1.4 ± 0.5 ^(defg)^
WPMm4	72.2 ± 11.1 ^(ab)^	3.3 ± 0.8 ^(cdefg)^
WPMm5	77.8 ± 5.5 ^(a)^	4.2 ± 0.9 ^(bcde)^
WPMm6	50 ± 25.4 ^(abc)^	2.1 ± 0.7 ^(defg)^

* Number of MB slices with at least one shoot per total number of treated MB slices × 100. Values are means (n = 18) ± standard error (SE). Means in columns with different letters are significantly different according to Duncan test (*p* < 0.05).

**Table 2:** Regeneration efficiency and the percentage of vitrified shoots for MB slices cultured for four weeks on regeneration media containing different gelling agents.

Gelling Agent Concentration (g L^−1^)		Average Number of Regenerated Shoots/MB ± SE			Frequency of Vitrified Shoots (%) ± SE *		
		QL2	QL6	WPMm5	QL2	QL6	WPMm5
PA	7	6.7 ± 0.5 ^(ab)^	7.4 ± 1.3 ^(ab)^	4.4 ± 0.7 ^(b)^	82.6 ± 5.7 ^(b)^	98.5 ± 8.9 ^(a)^	59.2 ± 5.9 ^(cd)^
B&V	6	5.6 ± 1 ^(ab)^	8.9 ± 0.7 ^(a)^	7.8 ± 1.4 ^(a)^	53.7 ± 4.3 ^(d)^	64.3 ± 7.4 ^(c)^	22.8 ± 4.9 ^(f)^

PA, plant agar (Duchefa Biochemie, Italy); B&V, PAS1000, plant agar (B&V, Parma, Italy). * Number of vitrified shoots per total number of treated MB slices × 100. Values are means (n = 18) ± standard error (SE). Means with significant (*p* < 0.05) differences are indicated with different letters according to Duncan test.

**Table 3:** GFP expression efficiency of Hansen 536 MB slices after three and 12 weeks of culture on WPMm5 medium supplemented with different kanamycin concentrations, or without kanamycin selection.

Kan Concentration (μM)	Number of Weeks after Co-Culture	Mean Number of GFP Spot/Explant ± SE	GFP MB (%) ± SE *	Number of MB Slices with Lzs	GFP MB Lzs (%) ± SE **
0	3	1.17 ± 0.37 ^(b)^	26.67 ± 5.75 ^(c)^	12	20 ± 5.21 ^(ns)^
	12	0.82 ± 0.38 ^(A)^	6.67 ± 3.25 ^(A)^	2	3.33 ± 2.34 ^(NS)^
17	3	2.10 ± 0.33 ^(ab)^	63.33 ± 6.5 ^(a)^	15	25 ± 5.64 ^(ns)^
	12	0.78 ± 0.29 ^(A)^	13.33 ± 4.42 ^(A)^	4	6.67 ± 3.25 ^(NS)^
42	3	3.17 ± 0.24 ^(a)^	48.33 ± 6.27 ^(b)^	22	36.67 ± 6.27 ^(ns)^
	12	0.68 ± 0.36 ^(A)^	8.33 ± 3.60 ^(A)^	1	1.67 ± 1.67 ^(NS)^

* GFP MB (%), Percentage of MB slices showing GFP fluorescence, selected either by kanamycin and GFP screening or by GFP visual selection alone (0 μM Kan), after three and 12 weeks on culture media. ** GFP MB Lzs (%), Percentage of MB slices showing large zones of GFP expression per total number of *Agrobacterium* inoculated MB slices. Values followed by small letters (a, b, c) compare explants cultured at three weeks after co-culture, while capital letter (A, B, C) compare explants cultured at 12 weeks after co-culture. Values are means (n = 60) ± standard error (SE). Means in columns with different letters are significantly different according to Duncan test (*p* < 0.05). NS, not significant.

**Table 4:** Combination of basal salts and growth regulators used in the regeneration media.

Culture Media	Basal Salts	Growth Regulators (μM)			
		BAP	IBA	TDZ	NAA
QL1	QL	4.4	0.5		
QL2	QL	8.9	0.5		
QL3	QL	4.4		0.2	
QL4	QL	8.9		0.2	
QL5	QL	4.4			0.1
QL6	QL	8.9			0.1
DKW1	DKW	4.4	0.5		
DKW2	DKW	8.9	0.5		
DKW3	DKW	4.4		0.2	
DKW4	DKW	8.9		0.2	
DKW5	DKW	4.4			0.1
DKW6	DKW	8.9			0.1
WPMm1	WPMm	4.4	0.5		
WPMm2	WPMm	8.9	0.5		
WPMm3	WPMm	4.4		0.2	
WPMm4	WPMm	8.9		0.2	
WPMm5	WPMm	4.4			0.1
WPMm6	WPMm	8.9			0.1

QL, salts and vitamins of Quoirin and Lepoivre (1977) [69]; DKW, salts and vitamins of Driver and Kuniyuki (1984) [70]; WPMm, microsalts and vitamins of Murashige and Skoog (1962) [68] and modified macrosalts of Lloyd and McCown (1980) [71]: 0.65 mM CaCl_2_, 5.93 mM Ca(NO_3_) × 4H_2_O, 1.25 mM KH_2_PO_4_, 5.68 mM K_2_SO_4_, 1.50 mM MgSO_4_, 5 mM NH_4_NO_3_.
